# Effects of* Cordyceps sinensis* on the Expressions of NF-*κ*B and TGF-*β*1 in Myocardium of Diabetic Rats

**DOI:** 10.1155/2015/369631

**Published:** 2015-12-02

**Authors:** You-you Gu, Huan Wang, Su Wang, Hua Gao, Ming-cai Qiu

**Affiliations:** ^1^Department of Endocrinology, The Fifth Central Hospital of Tianjin, Tianjin 300450, China; ^2^Community Health Service Center of Hu Jiayuan Street, Binhai New District, Tianjin 300454, China; ^3^Graduate School of Tianjin Medical University, Tianjin 300070, China; ^4^Department of Endocrinology, General Hospital of Tianjin Medical University, Tianjin 300052, China

## Abstract

*Objective.* To investigate the effect of* Cordyceps sinensis* (CS) on the expressions of NF-*κ*B and TGF-*β*1 in myocardium of streptozotocin-induced diabetic rats.* Methods.* A total of 53 healthy male SD rats, mice age of 8 weeks and weight of 220 ± 20 g, were randomly divided into five groups by randomized block design: normal control group (*n* = 10), diabetic group (*n* = 10), low dose of CS group (*n* = 12; CS 0.6 g·kg^−1^·d^−1^), middle dose of CS group (*n* = 11; CS 2.5 g·kg^−1^·d^−1^), and high dose of CS group (*n* = 10; CS 5 g·kg^−1^·d^−1^). The diabetic models with tail intravenous injection by streptozotocin (45 mg·kg^−1^). Diabetic rats were sacrificed after 8 weeks; the expressions of NF-*κ*B and TGF-*β*1 proteins and mRNA in the cardiac muscle were determined by using immunohistochemistry staining and reverse transcription polymerase chain reaction (RT-PCR) method. The data were analyzed using one factor analysis of variance.* Result.* The expressions of NF-*κ*B and TGF-*β*1 proteins and mRNA in the cardiac muscle of diabetic rats were significantly raised (*P* < 0.05), which could be decreased by CS (*P* < 0.05).* Conclusions.* The changes on the expressions of NF-*κ*B and TGF-*β*1 in myocardium may be involved in the occurrence of diabetic cardiomyopathy (DC). CS may play its role on myocardial protection by regulating the expressions of NF-*κ*B and TGF-*β*1 in myocardium.

## 1. Introduction

Chronic complications of diabetes mellitus (DM) can drag in whole body organs. DC is the specific lesions of the diabetic cardiac injury, and the primary pathological features are cardiomyocyte hypertrophy, dissolution and destruction of muscle fibers, loss of muscle horizontal stripes, hyaline degeneration, interstitial fibrosis, and inflammatory cell infiltration; however, its mechanism is still unclear. Recent studies showed that immune response and low-grade inflammation was closely associated with DM and DC, and the generation of inflammatory factor was the result of immune response. Recent research has found that the abnormal expressions of NF-*κ*B and TGF-*β*1 may participate in the occurrence of DC [1, 2]. Previous evidence suggested that streptozotocin induced diabetic rats had multiorgan immune damage, and immunosuppressive therapy can reduce the immune organ damage [3]. Immunosuppressant has many side effects, such as hepatic and renal toxicity and elevated blood pressure, so we should actively seek an effective therapeutic schedule which has smaller side effects. CS, also called Dong Chong Xia Cao (winter worm summer grass) in Chinese, is one of the most valued herbs in traditional Chinese medicine [4]. Yang et al. [5] consider that CS have strong inhibitory effect to immune function on body. In our research, we used CS to intervene in streptozotocin-induced diabetic rats and observed the effects of CS on the expressions of NF-*κ*B and TGF-*β*1 in myocardium of diabetic rats, and whether CS have myocardial protection of diabetic rats was evaluated.

## 2. Materials and Methods

### 2.1. Experimental Material

A total of 53 healthy male SD rats, mice age of 8 weeks and weight of 220 ± 20 g, were purchased from Beijing Experimental Animal Center of Chinese Academy of Sciences (China). CS was provided by Jiangxi Jimin Trusted Pharmaceutical Co., Ltd. (China). CS was dissolved in distilled water, with concentration of 0.5 g/mL solution. Streptozotocin was purchased from Sigma company in the United States. Rabbit anti-rat NF-*κ*B, TGF-*β*1, and PV-6001 immunohistochemical detection kit were bought from Wuhan Boster Biological Engineering Co., Ltd. (China). Reverse transcription-polymerase chain reaction (RT-PCR) primer was compounded by Nanjing Genscript Biotechnology Co., Ltd. (China). NF-*κ*B primer: sense 5′-CTGC GACA GATG GGCT ACAC-3′; antisense 5′-GGAA CGAT ATGA TGGC CTTTC-3′; product length 361 bp. TGF-*β*1 primer: Sense 5′-ACCC TTCC TGCT CCTC ATGG-3′; antisense 5′-AGCG CACG ATCA TGTT GGAC-3′; product length 392 bp. *β*-actin primer: sense 5′-TAAA GGGC ATCC TGGG CTAC ACTG-3′; antisense 5′-TTAC TCCT TGGA GGCC ATGT AGG-3′; product length 199 bp.

### 2.2. Rat Diabetes Model

All rats were randomly divided into five groups by randomized block design: normal control group (*n* = 10), diabetic group (*n* = 10), low dose of CS group (*n* = 12; CS 0.6 g·kg^−1^·d^−1^), middle dose of CS group (*n* = 11; CS 2.5 g·kg^−1^·d^−1^), and high dose of CS group (*n* = 10; CS 5 g·kg^−1^·d^−1^). The diabetic models with tail intravenous injection by streptozotocin (45 mg·kg^−1^), and normal control rats were injected with 0.1 M sodium citrate buffer. Blood samples were taken 7 days after the injection of streptozotocin or sodium citrate buffer, and rats with glucose level >16.7 mmol/L were considered diabetic. Normal control group and diabetic group used gavage with equivalent drinking water every day, and three CS groups used gavage with CS solution of different concentration. All rats were sacrificed after 8 weeks. The myocardium was separated and cut into two sections: the first section was fixed in 10% buffered formalin for hematoxylin eosin (HE) staining, Masson's trichome staining, and immunohistochemistry staining; the second section was stored at −80°C for RT-PCR.

### 2.3. HE Staining, Masson's Trichome Staining, and Immunohistochemistry Staining

HE staining, Masson's trichome staining, and immunohistochemistry staining used conventional paraffin embedding tissue and 5 *μ*m thick serial section. The ABC method was adopted to detect expressions of NF-*κ*B and TGF-*β*1 protein in myocardium. The dilute concentration of NF-*κ*B and TGF-*β*1 was 1 : 100 and 1 : 50. The position of positive reaction in image was analyzed with accumulative optical density value by the Image-Pro Plus 6.0.

### 2.4. RT-PCR

Tatal RNA 1 ug was prepared, and RNA was reverse transcribed into cDNA. NF-*κ*B reaction conditions were 94°C degeneration for 30 s, 67°C annealing for 30 s, 72°C extension for 40 s, and 35 cycles. TGF-*β*1 reaction conditions were 94°C degeneration for 30 s, 67°C annealing for 30 s, 72°C extension for 40 s, and 35 cycles. *β*-actin reaction conditions were 94°C degeneration for 40 s, 50°C annealing for 40 s, 72°C extension for 40 s, and 35 cycles. Amplification products were sequenced, and the images of agarose electrophoresis gel were acquired. The relative expression level of mRNA was calculated, and the calculating formula was as follows: the relative expression level of mRNA = absorbance values of objective stripe/absorbance values of *β*-actin.

### 2.5. Statistical Analysis

The data was represented in mean ± standard deviation. The data were analyzed using one factor analysis of variance by SPSS17.0. The *P* value < 0.05 was statistically significant.

## 3. Result

### 3.1. Pathological Features of Myocardium in Diabetic Rats

**Figure 1:**
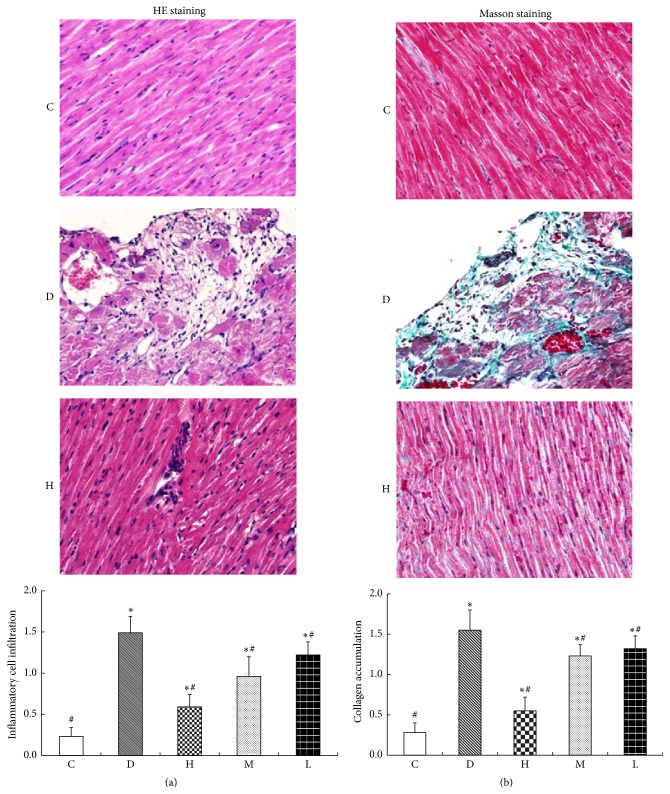
Pathological features of myocardium in diabetic rats. (a) HE staining; (b) Masson staining; C: normal control group; D: diabetic group; H: high dose of CS group; M: middle dose of CS; L: low dose of CS group. Compared with group C, ^*∗*^ *P* < 0.05; compared with group D, ^#^ *P* < 0.05.

The results of HE staining showed us that myocardial of normal control group rats had no obvious abnormalities. However, cardiomyocyte hypertrophy, myofiber dissolution and damage, loss of muscle horizontal stripes, and inflammatory cell infiltration were observed in myocardial of diabetic group (Figure 1). Masson's trichome staining revealed an increased collagen accumulation in myocardial intercellular substance and around blood vessels of streptozotocin-induced diabetic rats compared with normal control rats (Figure 1). Compared with diabetes group, quantification of inflammatory cell and collagen accumulation in the CS treatment groups were decreased (*P* < 0.05) (Figure 1).

### 3.2. NF-*κ*B and TGF-*β*1 Protein Expressions by Immunohistochemistry

**Figure 2:**
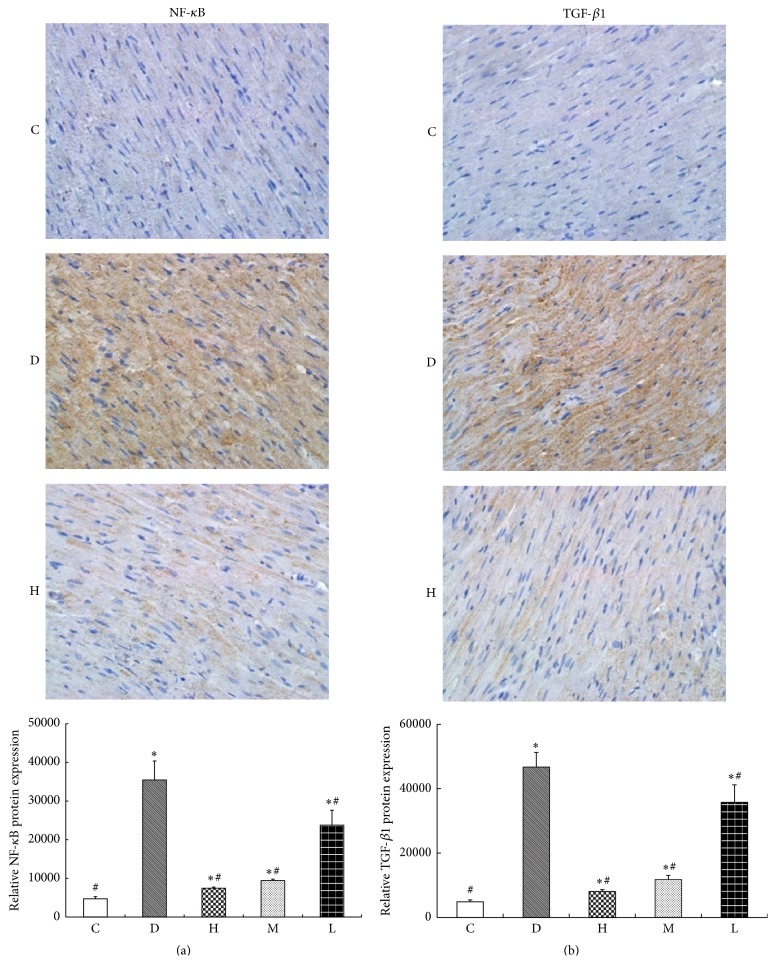
NF-*κ*B and TGF-*β*1 protein expressions by immunohistochemistry. (a) NF-*κ*B protein expression; (b) TGF-*β*1 protein expression; C: normal control group; D: diabetic group; H: high dose of CS group; M: middle dose of CS; L: low dose of CS group. Compared with group C, ^*∗*^ *P* < 0.05; compared with group D, ^#^ *P* < 0.05.

There was a little expression of the NF-*κ*B and TGF-*β*1 protein in myocardial cell of normal control group rats. The expressions of NF-*κ*B and TGF-*β*1 in myocardial cell of diabetic rats were significantly increased (*P* < 0.05) compared with normal control group rats. Compared with diabetes group, the expressions of NF-*κ*B and TGF-*β*1 in the CS treatment groups were decreased (*P* < 0.05), and the expressions of NF-*κ*B and TGF-*β*1 were reduced gradually with the increase of CS dose (Figure 2).

### 3.3. NF-*κ*B and TGF-*β*1 mRNA Expressions by RT-PCR

**Figure 3:**
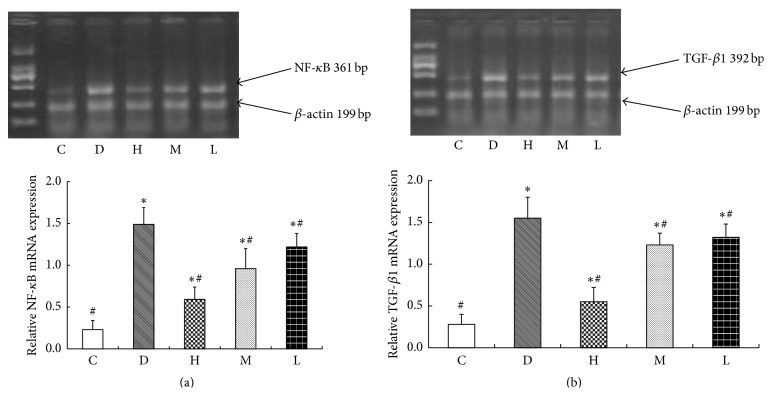
NF-*κ*B and TGF-*β*1 mRNA expressions by RT-PCR. (a) NF-*κ*B mRNA expression; (b) TGF-*β*1 mRNA expression; C: normal control group; D: diabetic group; H: high dose of CS group; M: middle dose of CS; L: low dose of CS group. Compared with group C, ^*∗*^ *P* < 0.05; compared with group D, ^#^ *P* < 0.05.

The amplification products of NF-*κ*B and TGF-*β*1 conformed to the gene sequence by sequencing analysis. In normal control group, a little expression of the NF-*κ*B and TGF-*β*1 mRNA was shown in cardiac muscle cell. Compared with normal control group, the expressions of NF-*κ*B and TGF-*β*1 were significantly increased in myocardial cell of diabetic rats (*P* < 0.05). The expressions of NF-*κ*B and TGF-*β*1 in the CS treatment groups were decreased compared with diabetes group (*P* < 0.05), and the expressions of NF-*κ*B and TGF-*β*1 were reduced gradually with the increase of CS dose (Figure 3).

## 4. Discussion

DC is the specific lesions of the diabetic cardiac injury, and the primary pathological features are cardiomyocyte hypertrophy, dissolution and destruction of muscle fibers, loss of muscle horizontal stripes, hyaline degeneration, interstitial fibrosis, and inflammatory cell infiltration. So far, DC pathogenesis has not been fully elucidated. Glucose and lipid metabolism disorder, insulin resistance, oxidative stress, apoptosis, microangiopathy, and myocardial fibrosis are involved in the occurrence and development of DC [6]. Recent studies showed that immune response and low-grade inflammation was closely associated with DM and DC, and the abnormal expressions of NF-*κ*B and TGF-*β*1 may participate in the occurrence of DC [1].

NF-*κ*B is a key transcription factor which was first found in B lymphocyte. NF-*κ*B can participate in many pathological and physiological processes by specific binding with enhancer sequence of immunoglobulin kappa light chain gene, such as immune response, cell apoptosis, and effect that cause cancer and inflammation. The NF-*κ*B almost exists in all types of tissues and cells and participate in a variety of signal transduction of inflammatory reaction [7]. The advanced glycation end products increased in diabetes patient's body, which combined with specific receptors on the cell membrane. Then they can release a large number of reactive oxygen species, prompt the NF-*κ*B, enter into the nucleus, start inflammation factors transcription, lead to the proliferation of vascular endothelial cells and smooth muscle cells, and promote the occurrence of DC. Animal experiments found that the activation of the NF-*κ*B can lead to increased oxidative stress, mitochondrial dysfunction and cardiac insufficiency in diabetic rats [8]. Studies from Wu et al. [9] showed that estrogen can inhibit fibroblast differentiation and proliferation which induced by angiotensin *П* through mitogen activated protein kinase (MAPK)/NF-*κ*B pathway, thus inhibiting myocardial fibrosis. These studies have indicated that the NF-*κ*B pathway was involved in the occurrence and progress of myocardial fibrosis.

TGF-*β*1, composed of two polypeptide chains, is a kind of growth factor which has a variety of biological activities. Studies have shown that abnormal expression and activity of TGF-*β*1 may be involved in the occurrence and development of DC. Hyperglycemia can induce increased synthesis of diacylglycerol pyrophosphate, activate protein kinase C, and result in the expression of TGF-*β*1 and significantly increased activity [10]. TGF-*β*1 may stimulate increased synthesis of cardiac fibroblasts I and III type collagen fiber and fibronectin and cause extracellular matrix obviously increased and extracellular matrix degradation reduced. Sun et al. [11] report that TGF-*β*1 may promote fibroblasts hyperplasia of differentiation and increase collagen synthesis and inhibition of collagen enzyme release in vitro cultures, and the expression of TGF-*β*1 increased significantly in fibrosis area with increased muscle fibroblast cells and macrophages.

In this study, immunohistochemistry and RT-PCR analysis indicated that expressions of NF-*κ*B and TGF-*β*1 increased in diabetic rats myocardial cells and suggest that the expression level changes of NF-*κ*B and TGF-*β*1 may be involved in the occurrence and development of DC.

CS is a kind of valuable Chinese traditional medicine which is rich in polysaccharides, amino acids, fatty acid, mannitol, and trace element. CS presents irreplaceable advantages from modern chemical synthetic drug, along with the further discussion of microscopic mechanism of prevention and treatment of diabetes. CS may play the role of myocardial protection by inhibiting aldose reductase and reducing inflammatory reaction and relieving immune complex deposition, resisting oxidation, and adjusting the immune function [8]. Different studies have different results for the influence of CS on the expressions of NF-*κ*B and TGF-*β*1. Wang et al. [12] reported that* Cordyceps sinensis* polysaccharide CPS-2 could inhibit PDGF-BB-induced human mesangial cells proliferation in a dose-dependent manner and return expression of PDGFR*β* and TGF-*β*1. Meng et al. [13] found that UM01, QH11, BNQM, GNCC, and DCXC are* Cordyceps* aqueous extracts, and UM01 significantly increased the expression of p-NF-*κ*B-p65 in a concentration dependent manner, while DCXC, GNCC, QH11, and BNQM had no obvious effects on expression of p-NF-*κ*B-p65 in RAW 264.7 macrophages. Yan et al. [14] considered that an acid polysaccharide fraction from* Cordyceps sinensis* fungus could increase the expressions of TNF-*α* and IL-12 and reduce the expression of IL-10 of Ana-1 cells and convert M2 macrophages to M1 phenotype by activating NF-*κ*B pathway.

In our topic, we observed cardiac protection of CS in diabetic rats, and used CS of low dose (0.6 g·kg^−1^·d^−1^), middle dose (2.5 g·kg^−1^·d^−1^), and high dose (5 g·kg^−1^·d^−1^) to intervene in the treatment of the diabetic rats induced by streptozotocin. Results showed that myocardial expressions of NF-*κ*B and TGF-*β*1 were decreased after CS treatment, and all three kinds of CS dose can reduce expressions of NF-*κ*B and TGF-*β*1. And the expressions of mRNA and protein showed a trend of gradual decline, along with the increase of CS dosage. Large dose of CS intervention can significantly reduce expressions of NF-*κ*B and TGF-*β*1 in myocardium of diabetic rats. We found that larger doses of CS intervention were likely to have more significant treatment effect in a relatively safe dose range. The occurrence of DC was the result of a variety of factors; the increased expressions level of NF-*κ*B and TGF-*β*1 may be only one of the taches. As a consequence, we will test the expressions of inflammatory and fibrogenesis related cytokines such as TNF-*α*, IL-1, IL-6, and type I Collagens I and III by experiment in our future, in order to further clarify the damage mechanism of DC and whether CS have an effect on its intervention.

In conclusion, CS intervention treatment can obviously reduce the myocardial injury of streptozotocin-induced diabetic rats. CS may play its role on myocardial protection by decreasing the expressions of NF-*κ*B and TGF-*β*1 in the myocardium, but the specific mechanism needs further study.
